# Evaluating minority representation across health care settings in hidradenitis suppurativa and psoriasis

**DOI:** 10.1097/JW9.0000000000000129

**Published:** 2024-01-18

**Authors:** Charlotte Greif, Ruby S. Gibson, Alexa B. Kimball, Zachary E. Holcomb, Martina L. Porter

**Affiliations:** a Preliminary Internal Medicine Program, Internal Medicine Department, University of Texas Health Science Center at San Antonio, San Antonio, Texas; b Tulane Dermatology Program, Dermatology Department, Tulane University School of Medicine, New Orleans, Louisiana; c Dermatology Department, Harvard Medical School, Clinical Laboratory for Epidemiology and Applied Research in Skin, Boston, Massachusetts; d Department of Dermatology, Beth Israel Deaconess Medical Center, Boston, Massachusetts; e Department of Medicine, Carilion Clinic, Section of Dermatology, Roanoke, Virginia; f Dermatology Department, Virginia Tech-Carilion School of Medicine, Roanoke, Virginia

**Keywords:** diversity, equity, hidradenitis suppurativa, minority, psoriasis, race

## Abstract

**Background:**

Females and minorities have been underrepresented in clinical research despite legislative efforts, including in hidradenitis suppurativa (HS) and psoriasis (PsO) clinical trials.

**Objective:**

To identify differences in demographic breakdowns of HS and PsO patients between health care settings to uncover any causative health disparities.

**Methods:**

This study reports racial, ethnic, and sex of HS and PsO patient populations across the emergency department (ED), inpatient, clinical trial, and registry settings. In addition, 95% confidence intervals are used as proxies of statistical significance to compare demographics between settings.

**Results:**

Female, Hispanic, and Black patients were underrepresented in HS clinical trials compared to their population prevalence (female: 63.7% vs 73.5%; Hispanic: 3.8% vs 12.0%; Black: 9.1% vs 20.3%). Female and Black patients were underrepresented in PsO trials compared to their population prevalence (female: 33.0% vs 54.8%; Black: 2.2% vs 5.7%). Black patients were overrepresented in the inpatient and ED settings in HS (inpatient vs ED vs population prevalence: 49.9% vs 49.9% vs 20.3%) and in the inpatient setting in PsO (inpatient vs population prevalence: 19.8% vs 5.7%).

**Limitations:**

The main limitation is the reliability and generalizability of the published studies used to compare demographics across settings.

**Conclusion:**

Underrepresentation of females and minorities in HS and PsO clinical trials is consistent with published literature. Overrepresentation of Black patients in acute care settings is likely multifactorial.

**What is known about this subject in regard to women and their families?**

- Females have historically been underrepresented in clinical research.
- Underrepresentation of females in clinical research may result in less effective and safe therapies for this population.

**What is new from this article as messages for women and their families?**

- Females are underrepresented in clinical trials evaluating systemic treatments for hidradenitis suppurativa and psoriasis.
- Underrepresentation of females may be due to barriers to trial enrollment, such as birth control requirements and no plans for pregnancy or breastfeeding.
- Better representation of females in clinical trials may result in more effective and safer therapies for females with hidradenitis suppurativa and psoriasis.

## Introduction

Hidradenitis suppurativa (HS) and psoriasis (PsO) are common chronic inflammatory skin conditions with an estimated global prevalence of <1 to 4% and 0.91 to 8.5%, respectively. Like many diseases, the predilection for developing HS and PsO differs across racial, ethnic, and sex groups.^1–4^ Ensuring clinical trial populations reflect the racial, ethnic, and sex makeup of patient populations is important for developing safe and effective drugs for all patient groups, as well as the formation of appropriate resources for patient education and support.^3^ Minorities are at a disproportionately high risk for disparities in treatment response and access to therapy compared to the general population. For example, minority patients demonstrated lower likelihood of responding to biologic treatment for psoriasis within 1 year of medication initiation compared to White patients, and variant *BCL2* gene polymorphisms by ethnicity have been shown to modulate response to adalimumab in HS patients.^3^ These and other environmental/genetic factors that may vary between demographic groups play a significant role in disease pathogenesis and response to therapy, highlighting the need for adequate representation of all demographic groups in medical research.

Available data indicate that Black, Hispanic, and female patients have historically been underrepresented in clinical trials, the impact of which may be underestimated given that race/ethnicity data is reported in less than one-third of published studies in high-impact journals across specialties.^5^ To remedy this, several pieces of legislation related to clinical trial representation and reporting have been introduced over the past 3 decades. In 1993, the National Institutes of Health passed a law promoting the inclusion of women and minorities in federally funded clinical research.^6,7^ In 2014, the Food and Drug Administration (FDA) developed an action plan to increase diversity in clinical trials and improve race/ethnicity reporting through publication of a “Drug Trials Snapshot,” which included breakdowns of sex, race, and age of clinical trial participants for newly FDA-approved drugs.^6,8^ In 2017, the Health Revitalization Act required all National Institutes of Health-funded research to report race and ethnicity, and the FDA currently requires all investigational new drug applications to report participant demographics for consideration of approval.^3,9,10^

Given the desirability of adequately representing racial, ethnic, and sex groups in clinical trials, it is helpful to determine the progress made from legislative action and increased awareness to inform next steps.

## Materials and methods

To identify demographic disparities in patients with HS and PsO, we examined clinical trials and other health care settings in the United States. We identified race, ethnicity, and sex of HS and PsO patients in the (1) U.S. population, (2) emergency department (ED), (3) inpatient, (4) clinical trial, and (5) registry settings, as detailed below. PubMed and ClincialTrial.gov searches were conducted from February to March of 2022 to identify studies. Studies were excluded for one or more of the following reasons: they were not conducted in the population of interest; they did not contain race, ethnicity, and sex data; they were conducted outside the United States. We included articles reporting the most detailed demographic breakdowns on the largest populations. Please see Mendeley Supplementary Figure 1, http://links.lww.com/IJWD/A40) for a detailed overview of our selection process.

(1) General U.S. population: We searched PubMed with the terms “Hidradenitis Suppurativa” or “Psoriasis” and “Race” or “Ethnicity” and filtered for papers published in the last 5 years. We identified 62 studies in HS and 314 in PsO. Following the exclusion criteria above and selecting the studies with the largest patient populations, we chose the study by Kilgour et al.^11^ (13,885 HS patients) for HS and Armstrong et al.^4^ (378 PsO patients) for PsO.
(2) ED: We searched PubMed using terms “Hidradenitis Suppurativa” or “Psoriasis” and “Emergency Department” published in the last 10 years. This resulted in 41 unique studies in HS and 206 in PsO. After applying the exclusion criteria, we chose Taylor et al.^12^ for HS (383,000 ED visits for HS) and we found no appropriate study for PsO.
(3) Inpatient: PubMed search terms included “Hidradenitis Suppurativa” or “Psoriasis” and “Inpatient” published in the last 10 years. We identified 28 studies in HS and 144 in PsO. After applying the exclusion criteria, we chose the papers by Edigin et al.^13^ (21,065 hospitalizations for HS) and Hsu et al.^14^ (2,738 hospitalizations for PsO).
(4) Clinical trial: We used ClinicalTrials.gov website to identify trials for inclusion (search terms shown in Supplementary Figure 1, http://links.lww.com/IJWD/A40) with start dates from January 1, 2010 to January 1, 2020.^15^ We identified 14 studies in HS and 267 in PsO. We excluded all studies using topical rather than systemic therapy, studies that did not evaluate the efficacy of treatment, and studies in different indications than HS and PsO. We included 13 HS studies and 156 PsO studies.
(5) Registry: A registry study is an observational study used to gather information about a certain population.^16^ We searched PubMed for “Hidradenitis Suppurativa” or “Psoriasis” and “Registry” and “Baseline Characteristics.” We did not specify a window of time for our search, since doing so significantly limited our search results. We identified 4 studies in HS and 49 in PsO. After applying the exclusion criteria, we chose articles by Prens et al.^17^ (594 patients in the HS UNITE Registry) and Singh et al.^18^ (7,511 patients in the PsO PSOLAR Registry).

**Fig. 1.**
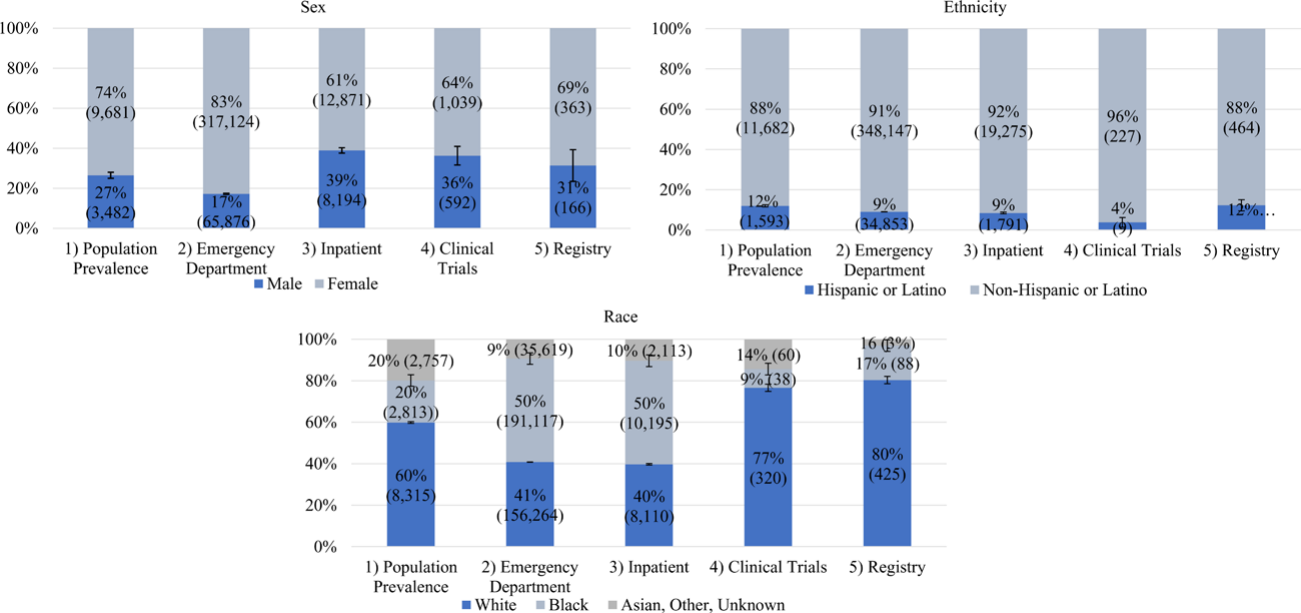
HS sex, ethnicity, and race by health care setting. Note: Error bars represent 95% confidence intervals. HS, hidradenitis suppurativa.

**Fig. 2.**
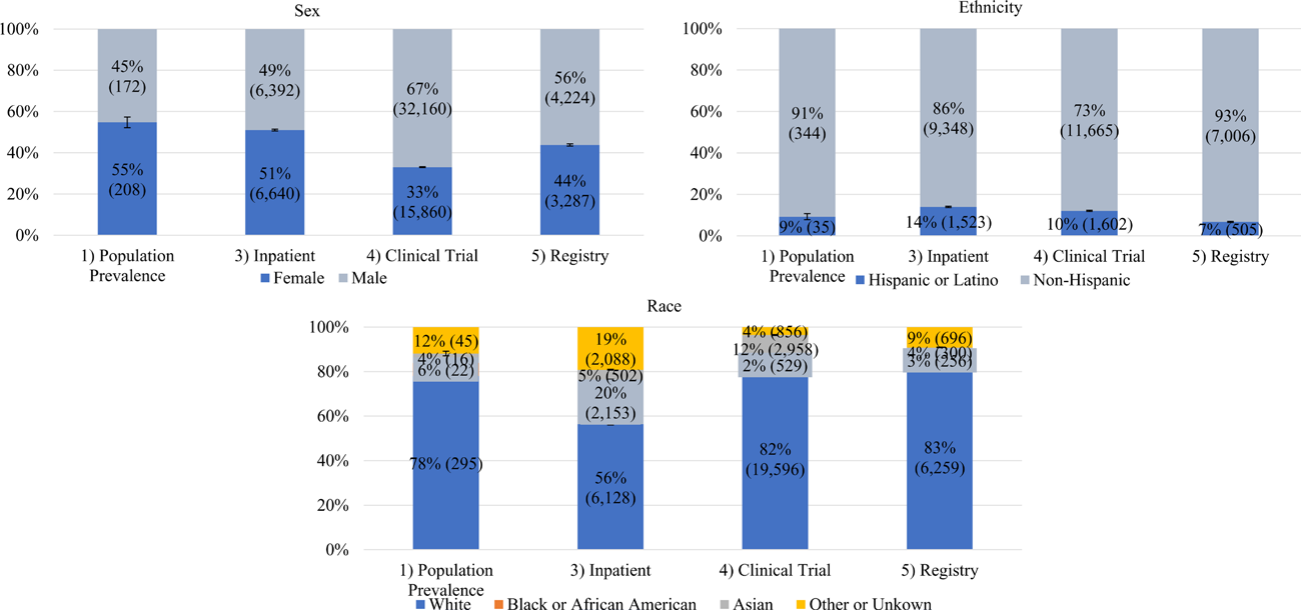
Psoriasis sex, ethnicity, and race by health care setting. Note: Error bars represent 95% confidence intervals.

We calculated 95% confidence intervals of demographic profiles and compared profiles across settings and diseases (Figs. 1 and 2, Tables 1 and 2).

**Table 1:** Hidradenitis suppurativa (HS) demographics by health care setting

Demographic characteristics *N* (% [95% confidence interval])	Population prevalence	Emergency department	Inpatient	Clinical trials	Registry
Data source	Kilgour et al.^11^	Taylor et al.^12^	Edigin et al.^13^	US National Library of Medicine^15^	Prens et al.^17^
Sex Female Male	9 681 (73.5% [72.8–74.3%]) 3,482 (26.5% [25.7–27.2%])	317,124 (82.8% [82.7–82.9%]) 65,876 (17.2% [17.1–17.3%])	12,871 (61.1% [60.4–61.8%]) 8,194 (38.9% [38.2–39.6%])	1,039 (63.7% [61.4–66.0%]) 592 (36.3% [34.0–38.6%])	363 (68.6% [64.7–72.6%]) 166 (31.4% [27.3–35.3%])
Ethnicity Hispanic Not Hispanic	1,593 (12.0% [11.4–12.6%]) 11,682 (88.0% [87.4–88.6%])	34,853 (9.1% [9.0–9.2%]) 348,147 (90.9% [90.8–91.0%])	1,791 (8.5% [8.1–8.9%]) 19,725 (91.5% [91.1–91.9%])	9 (3.8% [1.4–6.3%]) 227 (96.2% [93.7–98.6%])	65 (12.3% [9.5–15.1%]) 464 (87.7% [84.9–90.5%])
Race White Black Asian Other or unknown	8,315 (59.9% [59.1–60.7%]) 2,813 (20.3% [19.6–20.9%]) 442 (3.2% [2.9–3.5%]) 2,315 (16.7% [16.1–17.3%])	156,264 (40.8% [40.6–41.0%]) 191,117 (49.9% [49.7–50.1%]) Asian reported as other 35,619 (9.3% [9.2–9.4%])	8,110 (39.7% [39.1–40.4%]) 10,195 (49.9% [49.3–50.6%]) 169 (0.82% [0.7–1.0%]) 1,944 (9.5% [9.1–9.9%])	320 (76.6% [72.5–80.6%]) 38 (9.1% [6.3–11.8%]) 17 (4.1% [2.2–6.0%]) 43 (10.3% [7.4–13.2%])	425 (80.3% [77.0–83.7%]) 88 (16.6% [13.5–19.8%]) 11 (2.1% [0.9–3.3%]) 5 (0.9% [0.1–1.8%])

**Table 2:** Psoriasis (PsO) demographics by health care setting

Demographic characteristics *N* (% [95% confidence interval])	Population prevalence	Inpatient	Clinical trials	Registry
Source of data	Armstrong et al.^4^	Hsu et al.^14^	US National Library of Medicine^15^	Singh et al.^18^
Sex Female Male	208 (54.8% [49.8–59.8%]) 172 (45.2% [40.2–50.2%])	6,640 (51.0% [50.1–51.8%]) 6,392 (49.1% [48.2–49.9%])	15,860 (33.0% [32.6–33.5%]) 32,160 (67.0% [66.6–67.3%])	3,287 (43.8% [42.6–44.9%]) 4,224 (56.2% [55.1–57.4%])
Ethnicity Hispanic/Latino Not Hispanic/Latino	35 (9.3% [6.4–12.2%]) 344 (90.7% [87.8–93.6%])	1,523 (14.0% [13.4–14.7%]) 9,348 (86.0% [85.3–86.6%])	1,602 (10.1% [7.3–12.8%]) 11,665 (73.2% [72.5–73.8%])	505 (6.7% [6.2–7.3%]) 7,006 (93.3% [92.7–93.8%])
Race White Black Asian Other or unknown	295 (78.0% [73.8–82.2%]) 22 (5.7% [3.4–8.0%]) 16 (4.3% [2.3–6.3%]) 45 (12.0% [8.7–15.3%])	6,128 (56.4% [55.4–57.3%]) 2,153 (19.8% [19.1–20.6%]) 502 (4.6% [3.8–5.4%]) 2,088 (19.2% [17.5–20.9%])	19,596 (81.9% [81.4–82.4%]) 529 (2.2% [2.0–2.4%]) 2,958 (12.4% [11.9–12.8%]) 856 (3.6% [3.3–3.8%])	6,259 (83.3% [82.5–84.2%]) 256 (3.4% [3.0–3.8%]) 300 (4.0% [3.6–4.4%]) 696 (9.3% [8.6–9.9%])

Additional analyses included determining reporting rates of race, ethnicity, and sex in clinical trials. We found that only 20% of HS and PsO trials with start dates from 2010 to 2015 reported race and ethnicity overall and that 53% of HS and PsO trials with start dates from 2015 to 2020 did so. These rates were lower than those found by Charrow et al.^19^ for 2010 to 2015 (59.8%) and Chen et al.^8^ for 2015 to 2022 (72%).

## Results

### Demographics of HS

We identified several demographic differences across settings in HS patients (Table 1, Fig. 1). In clinical trials, female (63.7%), Hispanic (3.8%), and Black (9.1%) patients were underrepresented compared to their population prevalence (female: 73.5%; Hispanic: 12.0%; Black: 20.3%). White (76.6%) and Asian (4.1%) patients were overrepresented in clinical trials compared to their population prevalence (White: 59.9%; Asian: 3.2%). Compared to population prevalence, Black patients were overrepresented in the ED (49.9%) and inpatient (49.9%) settings, while White patients were underrepresented in these same settings (ED: 40.8%; inpatient: 39.7%).

### Demographics of PsO

As demonstrated in Table 2 and Fig. 2, clinical trial representation of female (33.0%) and Black (2.2%) patients was significantly lower in PsO clinical trials compared to their respective population disease prevalence (female: 54.8%; Black: 5.7%). White (clinical trial: 81.9%; population prevalence: 78.0%) and Hispanic (clinical trial: 10.1%; population prevalence: 9.3%) patients were well represented in clinical trials, while Asian patients were overrepresented in this setting (clinical trial: 12.4%; population prevalence: 4.3%). White patients were underrepresented in the inpatient setting (56.4%) compared to population prevalence, while Black (19.8%) patients were overrepresented.

## Discussion

Historical disparities in diversity representation in clinical trials have been a major focus in recent years, and steps have been taken to increase awareness and improve underrepresented representation. However, these disparities continue to persist in both clinical trial and various health care settings across demographic groups for HS and PsO patients. Our data suggest that population prevalence and clinical trial representation do not match across several demographics, including underrepresentation of female, Black, and Hispanic patients.

The underrepresentation of females in both HS and PsO clinical trials is consistent with historical data in clinical research, the etiology of which seems multifactorial.^20^ In 1977, females of childbearing age were banned from clinical trials out of concern for the fetus, but over time their inclusion was increasingly viewed as important to developing safe and effective drugs.^12^ In 1993, the FDA rescinded this law and enacted several policies to encourage female inclusion in trials.^12^ Despite these efforts, female underrepresentation persists, and female clinical trial participants remain subject to stricter requirements, such as the use of 2 or more forms of contraception and the declaration of no plans for pregnancy or breastfeeding.^12,21,22^ In HS, a disease predominantly affecting females of childbearing age, these requirements may pose a significant barrier to participation and prohibitively exclude a crucial demographic.^22,23^ While awareness of the problem is a necessary first step, additional considerations are needed to improve clinical trial access for female patients. The major limitation of clinical trial enrollment seems to be pregnancy concerns, both by the patients and by strict requirements for enrollment. Despite enrollment conditions designed to prevent pregnancy, there continue to be many pregnancies in HS trials, calling into question whether these requirements achieve their aim.^22,23^ It may be beneficial to consider the likelihood of pregnancy and potential impact of the drug on the fetus. Allowing more leniency for female patients, such as exempting patients who are infertile or postmenopausal or those whose exclusive sexual activity is with same-sex partners, seems like a reasonable step to increase clinical trial availability for more female patients. Females over the age of 40 may be another group appropriate for lenient requirements, as they are not good candidates for birth control and less likely to become pregnant.

Hispanic and Black patients were also underrepresented in clinical research in our study, consistent with the previously published findings. Possible reasons include lack of awareness of trial opportunities, lack of transportation, distrust in research and the health care system, fear of medication side effects, and lack of time.^6,24^ Non-English-speaking Hispanic patients face an additional barrier. Some studies show approximately a quarter of Hispanic patients in the United States lack adequate English-speaking skills, and when taking into consideration medical literacy, the number of Hispanic patients who can make an informed decision to pursue a clinical trial is even lower.^25^ The FDA recommends translating informed consent forms and reviewing them orally with patients who do not speak English, but this takes time and translators can be scarce.^26^ A 2016 survey of 4,586 hospitals in the United States found that only 56% of hospitals offer translation services and many are costly.^27^ Increasing the number of Spanish-speaking dermatology investigators would potentially decrease the language barrier. Furthermore, clinical trials often exclude patients with comorbidities, contributing to underrepresentation of Hispanic and other minorities who are disproportionately affected by various diseases.^28,29^

In addition to lack of minority representation in clinical trials, our data suggest an overrepresentation of certain minority groups with HS or PsO in various acute health care settings, such as the ED or inpatient hospital setting. In this analysis, we compared ED and inpatient visits to patient population prevalence. Overrepresentation may partially be a result of the ability of one patient to make multiple trips to the ED or hospital. However, more visits in certain minority groups likely suggest worse control of disease. Black patients with HS and PsO had a disproportionately high rate of hospitalizations compared to overall prevalence of disease, and Black patients with HS were more likely to seek emergency care. While this disparity is likely multifactorial, part of this disproportionality may stem from lack of adequate research and treatment options. There are also socioeconomic factors at play, as recent studies have shown that Black patients are less likely to have health insurance or a physician they see regularly compared to White patients.^30–33^ These inequities arise in part from a system that can disadvantage and hinder minority populations from seeking care, and one step in remedying this is to make additional efforts to ensure appropriate clinical trial representation among all demographics, including across socioeconomic classes. Anecdotally, the authors of this manuscript have found that Black patients have different perceptions and hesitations toward joining clinical trials than White patients, motivating them to ask questions about response to treatment among other Black patients in the study, chances of receiving placebo, known adverse events, and compensation.

We also found suboptimal reporting rates of race and ethnicity, but these improve over time, consistent with prior data published by Charrow et al.^19^ and Chen et al.^8^ Reporting of race and ethnicity increased after implementation of the FDA Action Plan in 2014 and the Health Revitalization Act in 2017. This provides a sign of encouragement that a shift is occurring toward better reporting and representation for all demographics in clinical research. All HS and PsO clinical trials included in our study reported sex.

The main limitation is the reliability and generalizability of the published studies we used to compare demographics across settings. Other limitations include exclusion of trials investigating topical medications, skewing our patient populations toward more severe disease, and differences in standardization of reporting of race, ethnicity, and sex, including self-determination of racial and ethnic status. For example, the paper by Taylor et al.^12^ combined “Asian,” “other,” and “unknown” race categories, limiting our ability to analyze Asian patients across all health care settings in HS. An additional unaccounted confounding factor is the socioeconomic status of each demographic group, which may impact patients’ participation in clinical trials as well as their representation in various health care settings for their underlying disease.

In conclusion, female and Black patients continue to be underrepresented in HS and PsO clinical trials evaluating systemic therapy. There is a disproportionate representation in HS and PsO inpatient hospital admissions for Black patients. Addressing clinical trial demographic disparities may play a role in providing more effective therapies to all patients. Reporting on race and ethnicity in clinical trials has been increasing over time but remains imperfect and is not universal. More research and advocacy are needed to ensure adequate clinical trial representation for all demographic groups.

## Conflicts of interest

C.G. has no disclosures. R.S.G. receives fellowship funding from the National Psoriasis Foundation that goes directly to her institution and is an investigator for Novartis, Abbvie, UCB, Pfizer, Lilly, Incyte, Anaptys Bio, Moonlake, Aristea, Sonomo Bio, and Janssen. A.B.K. is a consultant and investigator for Novartis, Abbvie, UCB, Pfizer, Lilly, Incyte, Anaptys Bio, Moonlake, Aristea, Sonomo Bio, and Janssen. Her fellowship program receives funding from Janssen and Abbvie. Z.E.H. has no disclosures. M.L.P. serves as a consultant and/or investigator for AbbVie, Pfizer, UCB, Trifecta Clinical, Eli Lilly, Janssen, Incyte, Anaptys Bio, Moonlake, Aristea, Sonomo Bio, and Novartis.

## Funding

None.

## Study approval

N/A.

## Author contributions

CG and RSG contributed to the project design, data collection, and writing/editing of this manuscript. ZEH, ABK, and MLP contributed to the conceptualization of this project, the project design, and the manuscript writing/editing.

## Supplementary data

Supplementary material associated with this article can be found at http://links.lww.com/IJWD/A40.

## Supplementary Material
