# Supplementary material for: Evaluating minority representation across health care settings in hidradenitis suppurativa and psoriasis
Source: Int J Womens Dermatol. 2024 Jan 18;10(1):e129. doi: 10.1097/JW9.0000000000000129 (PMC10796135; doi:10.1097/JW9.0000000000000129)
Supplement: Supplementary file 1 [file jw9-10-e129-s001.pdf]

Figure 1: Study Selection Process

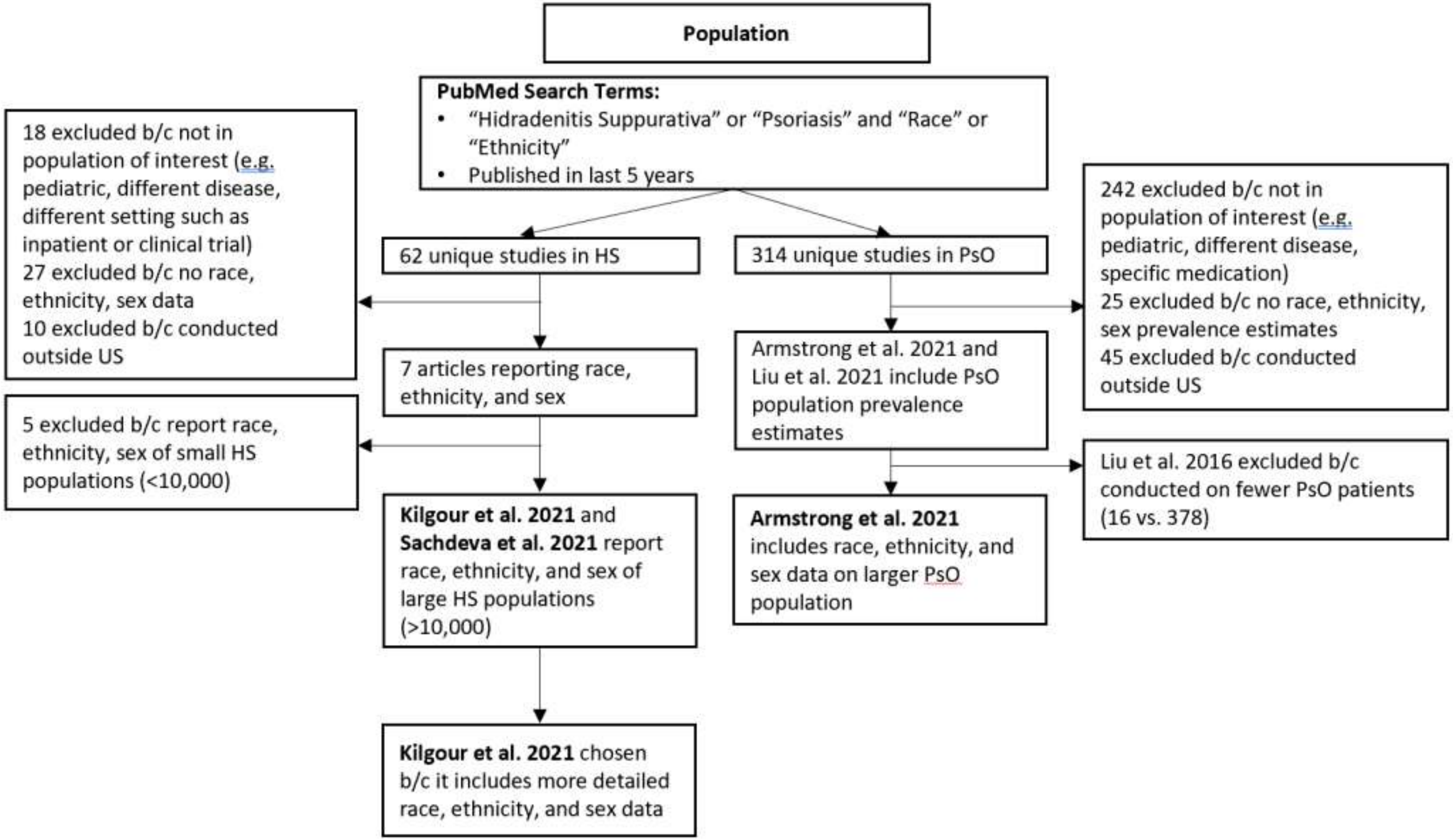

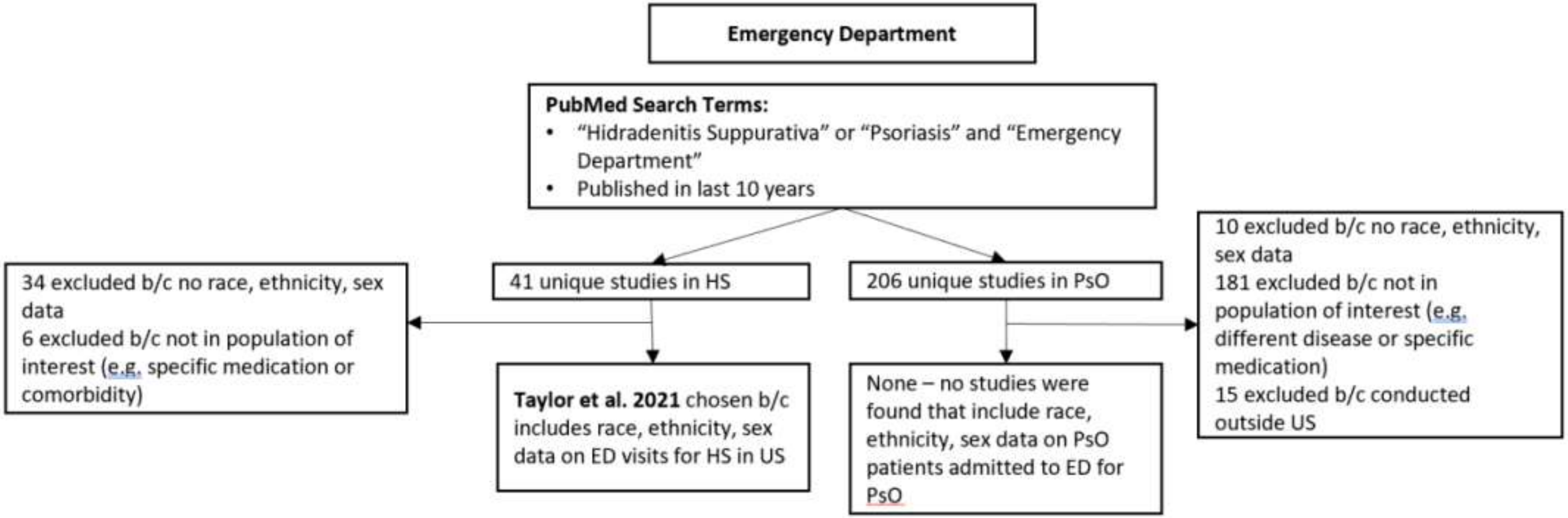
Emergency Department
PubMed Search Terms:
• "Hidradenitis Suppurativa" or "Psoriasis" and "Emergency Department"
• Published in last 10 years
41 unique studies in HS
206 unique studies in PsO
34 excluded b/c no race, ethnicity, sex data
6 excluded b/c not in population of interest (e.g. specific medication or comorbidity)
Taylor et al. 2021 chosen b/c includes race, ethnicity, sex data on ED visits for HS in US
None – no studies were found that include race, ethnicity, sex data on PsO patients admitted to ED for PsO
10 excluded b/c no race, ethnicity, sex data
181 excluded b/c not in population of interest (e.g. different disease or specific medication)
15 excluded b/c conducted outside US

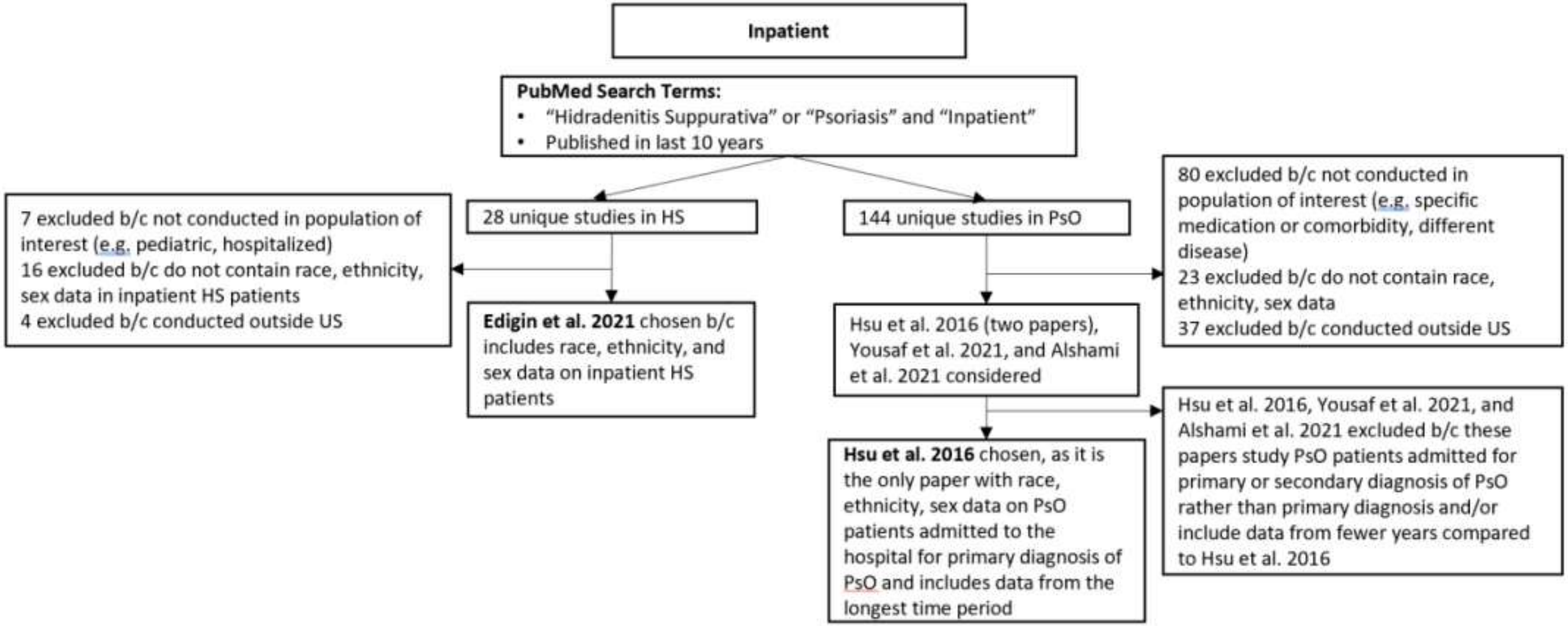
Inpatient
PubMed Search Terms:
• "Hidradenitis Suppurativa" or "Psoriasis" and "Inpatient"
• Published in last 10 years
28 unique studies in HS
144 unique studies in PsO
7 excluded b/c not conducted in population of interest (e.g. pediatric, hospitalized)
16 excluded b/c do not contain race, ethnicity, sex data in inpatient HS patients
4 excluded b/c conducted outside US
Edigin et al. 2021 chosen b/c includes race, ethnicity, and sex data on inpatient HS patients
80 excluded b/c not conducted in population of interest (e.g. specific medication or comorbidity, different disease)
23 excluded b/c do not contain race, ethnicity, sex data
37 excluded b/c conducted outside US
Hsu et al. 2016 (two papers), Yousaf et al. 2021, and Alshami et al. 2021 considered
Hsu et al. 2016, Yousaf et al. 2021, and Alshami et al. 2021 excluded b/c these papers study PsO patients admitted for primary or secondary diagnosis of PsO rather than primary diagnosis and/or include data from fewer years compared to Hsu et al. 2016
Hsu et al. 2016 chosen, as it is the only paper with race, ethnicity, sex data on PsO patients admitted to the hospital for primary diagnosis of PsO and includes data from the longest time period

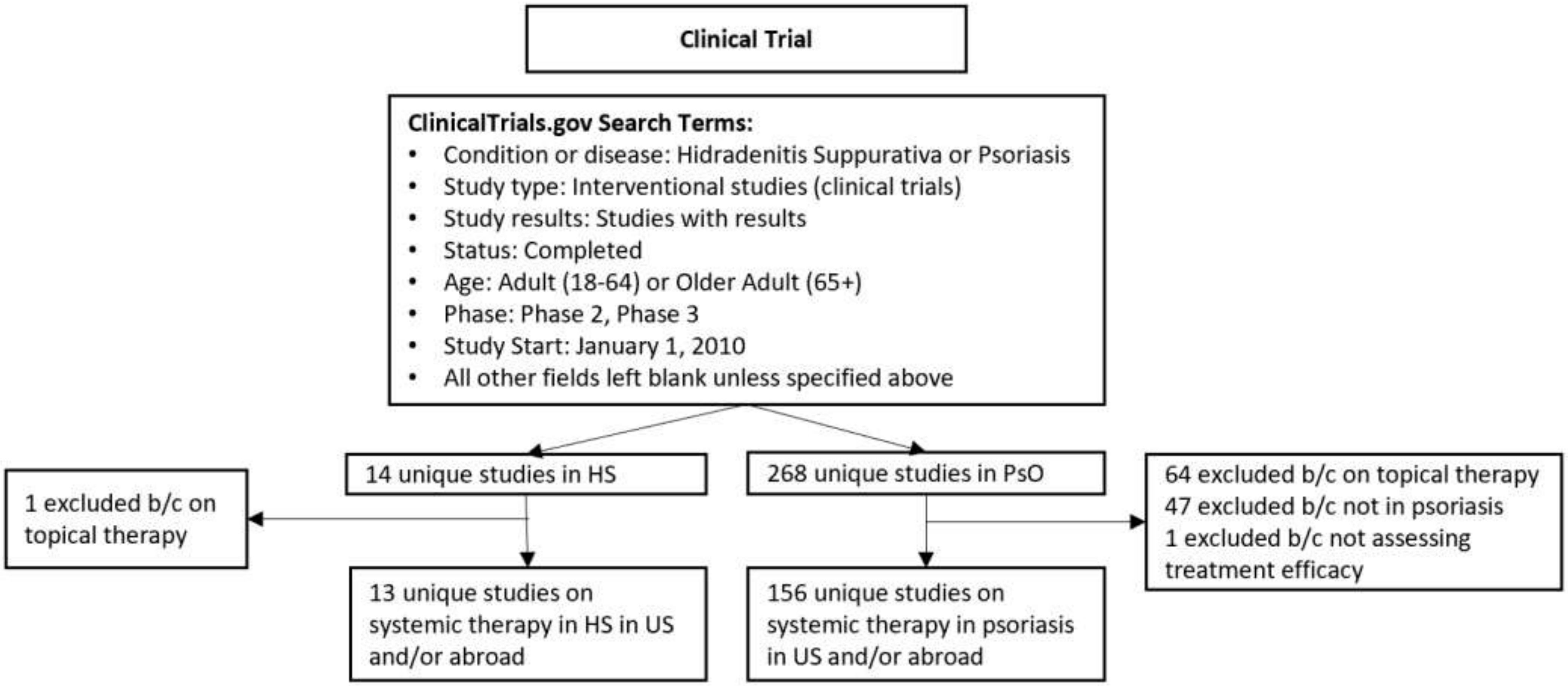
Clinical Trial
ClinicalTrials.gov Search Terms:
• Condition or disease: Hidradenitis Suppurativa or Psoriasis
• Study type: Interventional studies (clinical trials)
• Study results: Studies with results
• Status: Completed
• Age: Adult (18-64) or Older Adult (65+)
• Phase: Phase 2, Phase 3
• Study Start: January 1, 2010
• All other fields left blank unless specified above
14 unique studies in HS
1 excluded b/c on topical therapy
13 unique studies on systemic therapy in HS in US and/or abroad
268 unique studies in PsO
64 excluded b/c on topical therapy
47 excluded b/c not in psoriasis
1 excluded b/c not assessing treatment efficacy
156 unique studies on systemic therapy in psoriasis in US and/or abroad

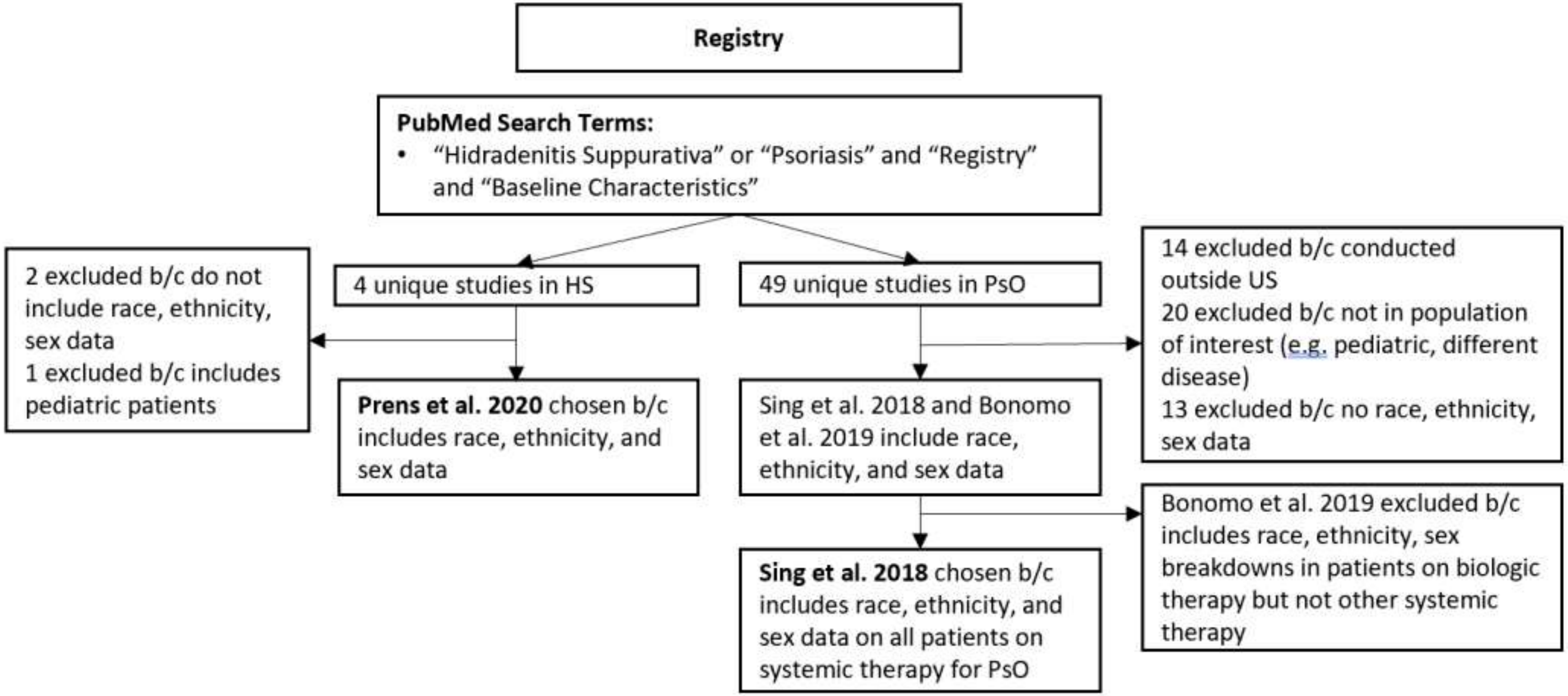
Registry
PubMed Search Terms:
"Hidradenitis Suppurativa" or "Psoriasis" and "Registry" and "Baseline Characteristics"
4 unique studies in HS
49 unique studies in PsO
2 excluded b/c do not include race, ethnicity, sex data
1 excluded b/c includes pediatric patients
Prens et al. 2020 chosen b/c includes race, ethnicity, and sex data
Sing et al. 2018 and Bonomo et al. 2019 include race, ethnicity, and sex data
14 excluded b/c conducted outside US
20 excluded b/c not in population of interest (e.g. pediatric, different disease)
13 excluded b/c no race, ethnicity, sex data
Bonomo et al. 2019 excluded b/c includes race, ethnicity, sex breakdowns in patients on biologic therapy but not other systemic therapy
Sing et al. 2018 chosen b/c includes race, ethnicity, and sex data on all patients on systemic therapy for PsO
